# Drug Hypersensitivity and Desensitizations: Mechanisms and New Approaches

**DOI:** 10.3390/ijms18061316

**Published:** 2017-06-20

**Authors:** Leticia de las Vecillas Sánchez, Leila A. Alenazy, Marlene Garcia-Neuer, Mariana C. Castells

**Affiliations:** 1 Division of Rheumatology, Immunology and Allergy, Department of Medicine, Brigham and Women’s Hospital, Harvard Medical School, Boston, MA 02115, USA; ldelasvecillassanchez@bwh.harvard.edu (L.d.l.V.S.); Leila_Alenazy@hms.harvard.edu (L.A.A.); Marlene_Garcia-Neuer@dfci.harvard.edu (M.G.-N.); 2 Department of Allergy, Marqués de Valdecilla University Hospital-IDIVAL, 39011 Santander, Spain; leticia.delasveci@gmail.com; 3 Department of Medicine, College of Medicine, King Saud University, Riyadh 12372, Saudi Arabia; 4 Master of Medical Sciences in Immunology Program, Harvard Medical School, Boston, MA 02115, USA

**Keywords:** drug hypersensitivity, desensitization, precision medicine, mast cells, desensitization models, high affinity IgE Fcε receptor I, IgE

## Abstract

Drug hypersensitivity reactions (HSRs) are increasing in the 21st Century with the ever expanding availability of new therapeutic agents. Patients with cancer, chronic inflammatory diseases, cystic fibrosis, or diabetes can become allergic to their first line therapy after repeated exposures or through cross reactivity with environmental allergens. Avoidance of the offending allergenic drug may impact disease management, quality of life, and life expectancy. Precision medicine provides new tools for the understanding and management of hypersensitivity reactions (HSRs), as well as a personalized treatment approach for IgE (Immunoglobuline E) and non-IgE mediated HSRs with drug desensitization (DS). DS induces a temporary hyporesponsive state by incremental escalation of sub-optimal doses of the offending drug. In vitro models have shown evidence that IgE desensitization is an antigen-specific process which blocks calcium flux, impacts antigen/IgE/FcεRI complex internalization and prevents the acute and late phase reactions as well as mast cell mediator release. Through a “bench to bedside” approach, in vitro desensitization models help elucidate the molecular pathways involved in DS, providing new insights to improved desensitization protocols for all patients. The aim of this review is to summarize up to date information on the drug HSRs, the IgE mediated mechanisms of desensitization, and their clinical applications.

## 1. Drug Hypersensitivity Reactions: New Clinical Approach Through Phenotypes, Endotypes, and Biomarkers

Drug hypersensitivity reactions (HSRs) are adverse effects of drugs [1,2]. Among the four most common HSRs described by Gell and Coombs, the most studied reactions are IgE (Immunoglobuline E)/mast cell mediated reactions which can cause cardiovascular collapse and anaphylaxis, leading to drug discontinuation which decrease quality of life and/or life expectancy [3,4,5,6].

**Figure 1:**
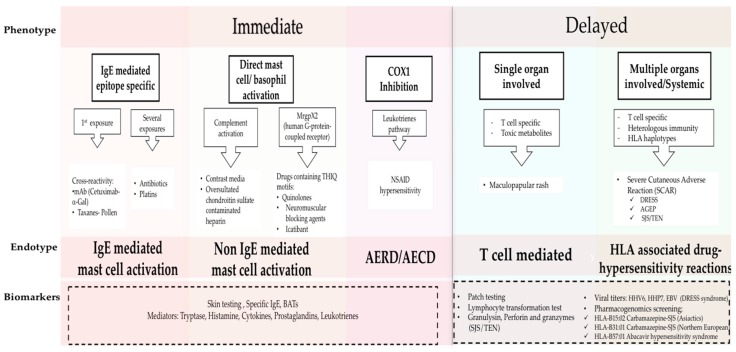
Phenotypes and Endotypes in drug allergy. The new classification of DHRs is based on phenotypes, endotypes and biomarkers. Phenotypes include immediate and delayed reactions; the clinical presentations of each phenotype are mediated by different immunological mechanisms which are defined by endotypes. Biomarkers are used to identify the Endotypes (dash line box). Adapted from Muraro, Antonella, et al. “Precision Medicine in Allergic Disease–Food Allergy, Drug Allergy, and Anaphylaxis-PRACTALL document of the European Academy of Allergy and Clinical Immunology and the American Academy of Allergy, Asthma & Immunology.” *Allergy* (2017) [1]. BAT, basophil activation test; mAb, monoclonal antibody; α-Gal, galactose-alpha-1,3-galactose; NSAID, nonsteroidal anti-inflammatory; AERD, Aspirin Exacerbated Respiratory Disease; AECD, Aspirin Exacerbated Cutaneous Disease; HHV 6, human herpesvirus 6; HHV 7, human herpesvirus 7; EBV, Epstein Barr Virus; DRESS, Drug reaction with Eosinophilia and Systemic Symptoms; AGEP, Acute Generalized Exanthematous Pustulesis; SJS-TEN, Stevens-Johnson Syndrome and Toxic Epidermal Necrolysis.

The classification of HSRs relies on the clinical presentation of typical symptoms and their timing [2,7], and were originally described by Gell and Coombs [8]: namely Type I (IgE mediated reactions), Type II (antibody mediated cytotoxicity reactions), Type III (immune complex-mediated reactions), and Type IV for delayed type hypersensitivity. Recently phenotypes, endotypes, and genotypes for these HSRs are being elucidated and applied to provide personalized approaches to treating and managing HSRs (Figure 1) [1]. Phenotypes in drug allergy focus on symptoms and timing, classifying the reactions as immediate or delayed, depending on the time between treatment administration and the onset of symptoms. Endotypes, based on cellular and biological mediators as well as biomarkers, have become vital to elucidate the molecular pathways as well as to evaluate the risk for reaction during re-exposure to the culprit drug [1,9,10,11,12]. Genetic predisposition has been shown to play a role in the development of HSRs to anticonvulsants, sulfonamides, and abacavir among others. Further investigation into pharmacogenetics will lead to prevention and better management of severe reactions such as Steven Johnsons Syndrome (SJS) and Drug Reaction with Eosinophilia and Systemic Symptoms (DRESS) [13,14]. A holistic understanding of drug HSRs can be achieved from combining classic and modern approaches: here we consolidate the new findings on molecular pathways with immediate (Type 1) and delayed (Type IV) phenotypes.

Cutaneous symptoms (like flushing, pruritus, or urticaria/angioedema), as well as respiratory and gastrointestinal symptoms are the most common mast cell activation related clinical presentations. More severe reactions with vital sign changes, throat tightness, or swelling can also appear [1,2,7,15]. Previous sensitization to the drug is usually required but cross reactivity has been described between drugs and other allergens (for example in pollen allergic patients who develop taxane hypersensitivity) [9]. Grading of the reaction severity is done with two classification systems: by Brown et al. [16] and modified Ring and Messemer [17] proposed criteria. HSRs are graded as mild/grade I (cutaneous symptoms or with only one symptomatic organ system), moderate/grade II (two or more systems involved without vital sign changes), and severe/grade III when more than two systems are affected with vital sign changes [16].

Reactions which occur during or within 1–6 h after the drug administration are classified as immediate reactions [1,2,8]. This phenotype typically includes the mast cell activation IgE mediated endotype, driven by epitope-specific IgE with mast cells as the main players. Other endotypes include direct complement activation [1,18], HSRs mediated by cyclooxygenase-1 inhibition in Aspirin Exacerbated Respiratory Disease (AERD) and Aspirin Exacerbated Cutaneous Disease (AECD) [19,20], or reactions due to some drugs with THIQ (tetrahydroisoquinoline)motifs which signal through the human G-protein-coupled receptor (MrgprX2) may also induce histamine release are included in the mast cell activation endotype [21,22]. In the past, symptoms such as fever, chills, and pain were not typically associated with allergic reactions, however, they have been reported during HSRs to monoclonals, oxaliplatin, and taxanes [3,6,9,23]. These type of reactions known as “cytokine storm-like reactions”, are mediated by the release of proinflammatory cytokines which activate macrophages and other immune cells with FcγR receptors [21].

Type IV reactions, classically known as delayed reactions, have a more heterogeneous presentation and typically appear several days or weeks after the exposure from the start date of drug administration [1,2,8]. They are related to T cell-mediated symptoms such as maculopapular exanthema or delayed urticaria and can also involve other organs such as liver, lungs, kidneys, or hematological alterations [1,8,13,24]. Severe Cutaneous Adverse Reactions (SCAR) which include Acute Generalized Exanthematous Pustulosis (AGEP), Drug Reaction with Eosinophilia and Systemic Symptoms (DRESS), Stevens-Johnson Syndrome (SJS), and Toxic Epidermal Necrolysis (TEN) have a severe clinical presentation with different treatment and poor clinical outcomes [13,14].

These phenotypes and endotypes can be further assessed through biomarkers such as skin testing (ST), and specific IgE and basophil activation tests (BAT), which help identify mast cell involvement in the HSRs as well as cross reactivity between drugs [7,9,10,11,25,26,27,28,29]. Levels of basophil/mast cell activation mediators during HSRs, such as tryptase and histamine, or cytokine and leukotriene production, may be helpful in identifying cells involved in the HSR. These mediators can also been usedin risk stratifying the patient [4,7,9,20,27,30,31]. In the last few years the relevance of genotypes in drug allergy has increased significantly; for example, specific HLA alleles have been associated with the development of hypersensitivity reactions to antibiotics, retrovirals, and anticonvulsant drugs [13,32]. Prescreening before abacavir treatment in HIV positive patients is required to identify potential reactors expressing HLA-B 57:01 [14]. To identify T-cell mediated hypersensitivity, diagnostic tools such as patch testing and lymphocyte transformation tests (LTT) have been proposed [1,24,33], and more recently, cytotoxic T-cell proteins such as granulysin, perforin, and granzyme B [34].

## 2. Drug Desensitization: A Revolutionary Approach to the Management of Type I and Type IV Drug Hypersensitivity Reactions

Drug desensitization (DS) was developed due to the pressing need to reintroduce drugs in a safe fashion in patients who had developed IgE/non IgE type I HSRs to critical antibiotics and/or other drugs. The first reported case was in 1942 in an English soldier in urgent need of penicillin at a time when no alternatives existed; further advances were made in the 1980s when the first oral and intravenous protocols for penicillin were created. The safety and efficacy of penicillin desensitization was widely described without reports of deaths or anaphylaxis to such a degree that it was even used in high risk populations such as pregnant women who had prick positive penicillin allergy and required treatment for syphilis (Table 1a) [35,36]. The first intravenous protocol was also reported with penicillin desensitization in 1987, and the protocol used a 10-fold escalation in solution concentration with 20 min intervals and was the prototype for modern desensitizations [37].

Desensitization today is indicated when patients have HSRs mediated by mast cell activation to their first line therapy without comparable alternatives [3,4,5,26,30,38,39]. Desensitization is achieved by incrementally escalating the sub-optimal doses of the culprit drug until the required dose is reached, and DS induces a temporary tolerance which protects the patient from anaphylaxis [6,23,38]. Currently valid DS protocols have been established for other chronic diseases such as Cystic fibrosis, which has poor patient prognosis if antibiotic restrictions are present [5]. Recently these procedures have been developed for new and innovative drugs for oncologic and chronic inflammatory diseases which were continued first line therapies that were critical for patients’ quality of life and life expectancy [3,4]. Previously published data have shown that more than 20% of oncology patients who receive platin chemotherapy developed an allergic reaction. Equally concerning is the increasing number of unique monoclonal antibody (mAbs) therapies which have high rates of immunogenicity due to non-human mAb parts and glycosylation [3]. Preventing patients from using first line therapy can be taxing both in terms of cost but also in the reduction of quality of life, life expectancy, and disease progression or management. In terms of safety and efficacy, previously published data have shown that DS is the best option when indicated. Successful protocols have been described for different HSRs to culprit drugs, for example antibiotics, biologics, chemotherapy, progesterone, as well as many other treatments [6,23,27,39,40,41]. A cost/efficacy analysis was also shown by Sloane et al. [6] indicating that DS does not increase health costs compared to standard treatment.

Understanding the molecular mechanisms of the HSR, patient’s comorbidities, skin testing, and genetic markers are critical to determine whether desensitization is indicated as well as the potential risk of reaction during the procedure.

DS has been established to be safe and effective for IgE mediated drug HSRs. Patients with immediate reactions to taxanes and other chemotherapies in which the IgE mechanism cannot be demonstrated have also been successfully desensitized [4,6]. HIV positive patients who present with delayed maculopapular exanthem have also been successfully desensitized, however protocols for delayed reactions have not yet been standardized [40]. DS is contraindicated in immune thrombocytopenia, serum sickness-like reactions, or SCAR (Severe Cutaneous Adverse Reactions) due to high toxicity [7,38,42]. Comorbidities, current prescriptions, and premedications should be evaluated to understand the initial reaction and appropriately risk stratify the patients because some medications may change the risk of reaction, treatment plan, or mask the severity of the initial symptoms. Skin testing and mediator levels are also key factors for evaluating the risk of reaction during desensitization and for understanding the original HSR [2,9,25,42]. Usually mild symptoms and negative skin tests are associated with lower risk of reaction, while moderate to severe reactions and/or positive skin prick tests indicate higher risk. This has been shown with the published taxane, carboplatin, and penicillin algorithms, however further research is necessary to validate skin testing with chemotherapies, monoclonals, and other drugs [9,12,27,28]. In vitro test results (such as specific IgE and basophil test activation) and pharmacogenetics have also been proposed to predict outcomes of DS [10,11,29]. Recent data has linked BRCA1/2 mutation in patients being treated with carboplatin with increased rates of IgE mediated HSRs [11,43]. These findings illuminate new insights in HSR prediction, management, and desensitization.

After risk stratification, a flexible protocol with 4 to 16 steps (typically 12) and escalating the dose 2 to 2.5 times every 15 min is applied. Usually the starting concentration of the solution in a 4 bags/16 steps protocol is 1/1000 to 1/100 in a 3 bags/12 steps protocol, reaching the target dose at the end of the procedure (Table 1b) [3,5,6,23,36,42,44].

Recently, algorithms to manage HSRs through DS have been described for antibiotics and chemotherapies [9,12,25,27], to allow some patients to tolerate the offending drug via regular infusion without safety concerns [9]. Other algorithms, such as that for platins, have focused on repeated skin testing to avoid false negative results [12,25].

While empiric desensitization protocols were established clinically to treat patients in need, the mechanisms of desensitization were evaluated in vitro to understand the cellular and moelcular players. The first in vitro studies occurred in animals; later, human basophils were used for proof of concept in vitro experiments by performing BAT (basophil activation test). Currently both in vivo and in vitro studies are used to understand the cellular and molecular pathways of mast cell and basophils involved in HSRs and anaphylaxis. A major step in improving clinical desensitization protocols was insight from in vitro studies on how dosing and timing during the desensitization protocol inhibited cell degranulation and cytokine production.

## 3. Mast Cells: Positive and Negative Regulation is Relevant to Desensitization

Mast Cells (MCs) are key effectors in many immune responses including IgE and non-IgE mediated HSRs and are believed to be the primary target cell in DS [45].

During HSRs, MCs can be activated through IgE or non-IgE dependent pathways. Recent studies have shown that small molecule drugs may induce systemic non-IgE mediated reactions through the activation of human MC G-protein coupled receptor (MrgprX2) [21,22]. The IgE mediated pathway is the one that is best defined: patients who are predisposed to developing an allergic reaction switch their allergen specific antibodies from IgM to IgE after several exposures. Subsequently, post immunoglobulin class switch, plasma cells produce a large amount of the specific allergen IgE which binds to high-affinity IgE Fc receptors (FcεRI) on basophils and mast cells. HSRs occur when a sensitized patient is re-exposed to the drug or during the first lifetime exposure with an already encountered allergen [9,46].

### 3.1. FcεRI Structure

FcεRI belongs to the multi-subunit immune receptor family and is constitutively expressed on human and mice MCs and basophils as a hetero-tetrameric receptor composed of an α, β, and two γ chains. In human dendritic cells and monocytes it is expressed as trimer αγ_2_. For allergen recognition, the α chain of the receptor binds to IgE through the two extracellular Ig-like domains on the Fc region of the antibody. The membrane-tetraspanning β chain and the two disulfide-linked identical γ chains contain a single immune-receptor tyrosine-based activation motif (ITAM) which is responsible for signal transduction. Phosphorylation of γ-subunit ITAMs is important in initiating and inducing downstream propagation of the intracellular signaling. On the other hand, phosphorylation of β-subunit ITAM has been suggested to have an amplifier and/or a suppressor function for the γ chain mediated signaling events. The Lyn tyrosine kinase is constitutively associated with the cytoplasmic tail of the FcεRI β chain [47,48,49].

### 3.2. Mast Cell Activation via FcεRI

FcεRI cross-linking, following allergen ligation to IgE-bound FcεRI, will trigger activation of the Lyn kinase. Activated Lyn initiates signal transduction through phosphorylation of the β and γ chain ITAMs. This leads to recruitment of Syk tyrosine kinase to phosphorylated γ chain ITAMs, Syk becomes activated and phosphorylates other enzymes and adaptor proteins to form multicomponent signaling cascade complexes. Phospholipase Cγ (PLCγ) recruits and binds to linker for activation of T cells (LAT), one of the adaptor proteins; PLCγ phosphorylates and then hydrolyzes phosphatidylinositol bisphosphate break down to yield inositol trisphosphate (IP3) and diacylglycerol (DAG). Two distinct downstream signaling pathways are mediated by the second messengers IP3 and DAG in MCs [46,47,48].

IP3 induces an increase in cytosolic calcium (Ca^2+^) concentration by binding to its receptor in the endoplasmic reticulum and rapidly inducing the “first phase” of calcium mobilization, by transient release of endoplasmic reticulum Ca^2+^ stores. STIM1 senses the depletion of endoplasmic reticulum Ca^2+^ stores and induces the opening of calcium release-activated calcium (CRAC) channels in the plasma membrane. Binding of STIM1 to ORAI, the pore-forming component of the CRAC channel, facilitates the entry of extracellular calcium into the cytosol. This leads to a prolonged Ca^2+^ influx, also known as “second phase” [47,49]. DAG activates protein kinase C (PKC), which is essential for inducing several cellular responses. In addition, Fyn tyrosine kinase, which phosphorylates Grb-2-associated binder-like protein 2 (Gab-2), activates PKC through the phosphoinositide 3-kinase (PI (3) K) pathway [46,47,48].

Both PKC activation and calcium flux play a critical role in the initiation of three main biological responses of MC activation: 1. Immediate release of an array of preformed biologically active inflammatory mediators including amines (histamine and serotonin), proteoglycans, neutral proteases (tryptase, chymases, and carboxypeptidases), β-hexosaminidases, cytokines, and growth factors (tumor necrosis factor α (TNFα,) and vascular endothelial growth factor (VEGF)) from the cytoplasmic granules, 2. An early de novo synthesis of lipid mediators from phospholipid metabolism and, 3. Late release of inflammatory cytokines [49].

### 3.3. Degranulation, Lipid Mediator, and Cytokine Production

The increase in cytosolic Ca^2+^ levels and the activation of PKC stimulate the degranulation machinery. Activated PKC phosphorylates the myosin light chain of cortical actin-myosin complexes, resulting in the disassembly of the complex and translocation of the cytosolic granules towards the plasma membrane in a microtubule-dependent manner [47]. As granules get closer to the plasma membrane they fuse through a process mediated by soluble *N*-ethylmaleimide-sensitive factor attachment protein receptor (SNAREs) proteins, and this allows the release of inflammatory mediators into the surrounding extracellular environment within a few minutes of crosslinking [50]. One of the major targets of activated extracellularpsignal-regulated Kinase (ERKs), a mitogen-activated protein kinase (MAPK), is the cytoplasmic PLA_2_. Once activated, PLA_2_ translocates into the cell membrane, where it hydrolyzes membrane phospholipids to release arachidonic acid, triggering leukotriene and prostaglandin formation [48,51]. Cytokine production occurs over 4–6 h after cross-linking due to the activation of several adaptor proteins required for the activation of nuclear factor-κB (NF-κB), nuclear factor of activated T-cells (NFAT), signal transducer and activator of transcription 6 (STAT-6), and activator protein 1 (AP-1) transcription factors which are crucial for the expression of many cytokine proteins, including IL-6 (interleukin 6), TNF-α, IL-1β and IL-13 [46,47].

### 3.4. Negative Regulation of Mast Cell Activation through FcεRI

MCs express a large number of inhibitory receptors; FcγRIIB (Fcγ receptor IIB), gp49B1/Leukocyte Immunoglobulin Like Receptor B (LILRB4), mast cell function-associated antigen (MAFA), paired Ig-like receptor (PIR)-B, and leukocyte-associated Ig-like receptor (LAIR) [52]. Immunoreceptor tyrosine-based inhibitory motif (ITIM) containing receptors downregulate MC activation through dephosphorization of multiple targets via the recruitment of various enzymes [49,52]. Colligation of cross-linked FcεRI to the inhibitory receptors will negatively regulate FcεRI signaling events through the recruitment of negative intracellular regulators. Lyn can initiate both activating and inhibitory signals, by phosphorylating the FcεRI ITAMs and inhibitory receptor ITIMs [47]. These events lead to inhibition of FcεRI-induced calcium flux which is required for early and late MC activation response.

Desensitized MCs show a complete abrogation of the phosphorylation of key IgE/FcεRI signaling components important for different stages such as Lyn, LAT, ERK, MAPK, and STAT-6 [45,53,54,55]. It is noteworthy that STAT-6 Knockout (STAT-6-KO)mouse bone marrow-derived mast cells (mBMMCs) failed to be desensitized which may suggest a possible role in inhibiting DS through a different pathway [55]. In vitro prolonged DS of human MCs and basophils showed a depletion of Syk, an activation signal transduction molecule, indicating a universal rather than specific desensitization [56,57]. It is not well understood how ITIM regulates ITAM equilibrium.

Many studies have suggested that agonist-induced internalization of G-protein coupled receptors (GPCRs) from the cell surface into intracellular compartments regulates cellular responsiveness [58,59].The multiple incremental suboptimal antigen doses administrated during DS may lead to the release of small amounts of histamine that would bind to receptors and subsequently induce internalization of the receptors. Thus the subthreshold release of MC mediators could be implicated in histamine receptor desensitization leading to MC hyporesponsiveness.

However there are studies which contradict the hypothesis of leukotriene receptor desensitization during DS. MC stimulation with a low concentration of leukotriene causes hyperresponsiveness to leukotriene stimulation, which can be attributed to the low concentration of leukotrienes leading to translocation of leukotriene receptors from the interior of the cell to the cell surface. Conversely, a high concentration of leukotriene will induce leukotriene receptor internalization and subsequently MC hyporesponsiveness to leukotriene stimulation [60].

## 4. Molecular Mechanisms in IgE Mast Cell Desensitization

### 4.1. Characterizing Desensitization Mechanisms through In Vitro and In Vivo Models

DS protocols have been shown to be safe and effective during re-exposure to the culprit drug[5,6,41,45,53,55]]. Highly allergic patients with IgE dependent reactions have presented with negative skin testing after desensitization, indicating inhibition of the mechanisms that induce mast cell activation [61].

To better understand how DS protocols allow patients to be treated repeatedly with the culprit drug after an IgE mediated HSR, several in vitro and in vivo models have been generated to provide support for this technique. An effective in vitro model of rapid IgE desensitization was developed using mBMMCs under physiologic calcium conditions and has subsequently been modeled into successful human DS protocols. By starting with subthreshold doses of the antigen (1/1000 or 1/100) and by administrating incremental doses of the antigen at fixed time intervals and increasing the dose 1.5 to 2.5 times at every step, sensitized mBMMCs are unresponsive through to the target dose [53,55].

**Figure 2:**
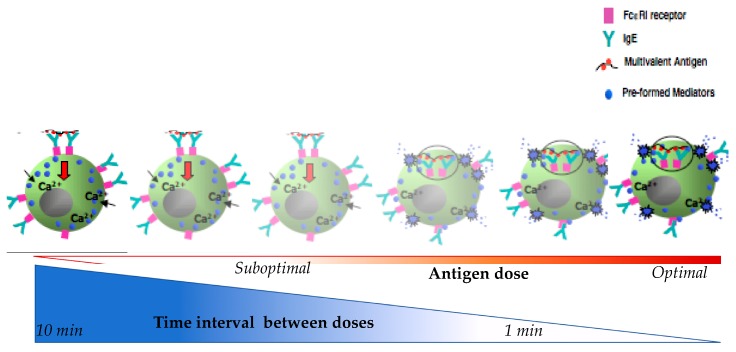
Schematic diagram represent of mast cells β-hexosaminidase in relation to time interval between doses and antigen doses during desensitization.

As shown in Figure 2, shorter intervals of antigen delivery and/or too high suboptimal antigen dosing leads to increased β-hexosaminidase release [55]. In this model when suboptimal doses are delivered at 1 min there is a littile inhibition of the mediators released but when delived at 10 min intervals, there is maximal inhibition. Regarding the antigen dose, suboptimal doses will trigger little releases as compare to optimal doses. This indicates that both the time and dose are critical to MC degranulation. The time interval length is inversely correlated with the amount of β-hexosaminidase release and may reflect the breakthrough reactions during human DS when the drug is delivered too quickly. Concurrently, the dose is directly correlated with the release of β-hexosaminidase and starting DS with a dose above the suboptimal threshold may cause a breakthrough reaction.

**Figure 3:**
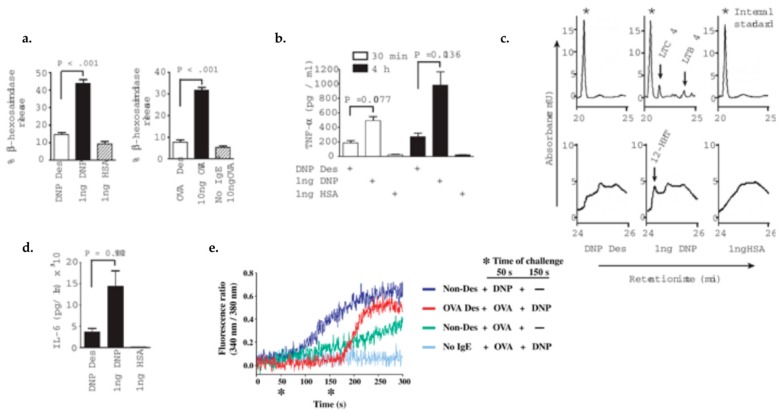
Mast cell IgE/Antigen desensitization inhibits the pre-formed mediators release, lipid mediators and cytokine production, and calcium flux and is antigen specific. 1. Pre-formed mediators release: (**a**) β-hexosaminidase release: MCs desensitized to DNP-HAS and OVA showed a 78% and 71% reduction of beta-hexosaminidase release, respectively compared to activated MCs with same allergen; (**b**) Pre-formed TNF-(white bars) and de novo synthesized TNF-(black bars) have a 62% and 75% reduction after desensitization, respectively compared with activation. 2. Lipid mediators production: (**c**) Arachidonic metabolites represent by Cysteinyl leukotriene C4 (LTC4), leukotriene B4 (LTB4), and 12(s)-hydroxyheptadeca-5Z, 8E, 10E-trienoic acid (12-HHT) were detected in MC supernatant after activation but not in control or desensitized MCs. 3. Cytokines production: (**d**) During DS, IL-6 production is 75% less than during activation. 4. calcium flux: (**e**) Calcium flux is impaired in OVA desensitized MCs after being triggered with activating dose of OVA but the influx is restored by activating with DNP-HAS non-desensitizing antigen (red line). Adapted from Sancho-Serra, et al., 2011 [53] with permission from John Wiley and Sons.

**Figure 4:**
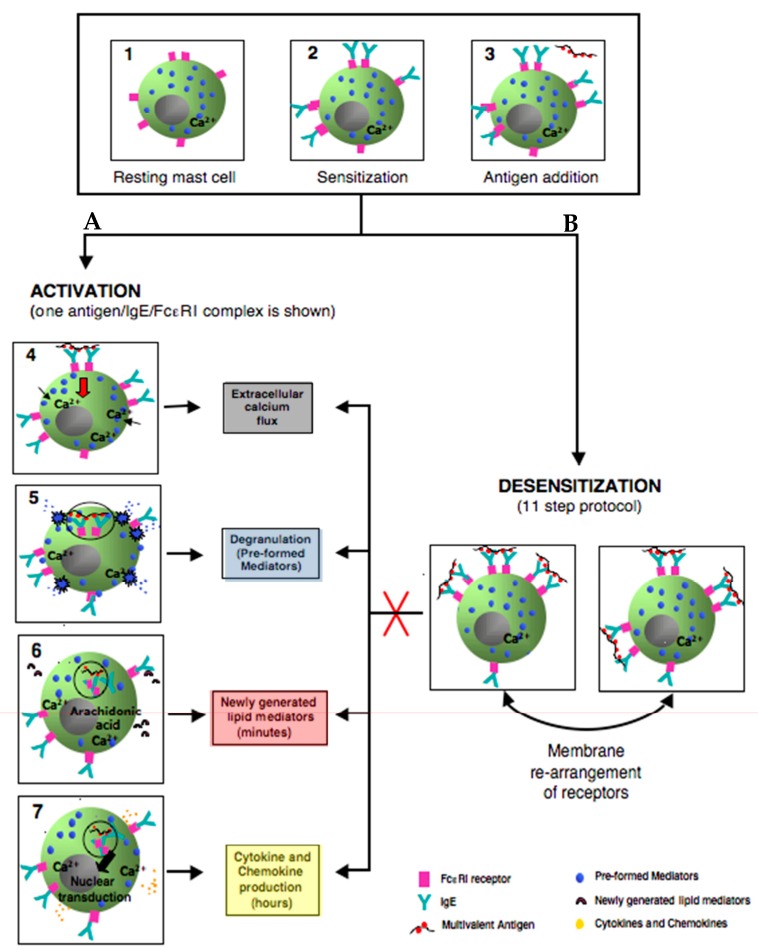
Simplified model comparing the outcomes of activated and rapidly desensitized mast cells. Resting mast cells (1) sensitizes with antigen-specific IgE bound to their high affinity IgE recepors (Fc*ε*RI) on cell surface (2) A: following the crosslinking of IgE/Fc*ε*RI receptors with an optimal dose of antigen leads to calcium flux (3–4), degranulation with release of the preformed mediators (5), early de novo synthesis of lipid mediators (6), and late cytokines/chemokines production(7). B: following administrating of suboptimal 11 doses to complete the same optimal dose as in A (activation) (3); leads to membrane rearrangements of receptors which impairs Ag/IgE/Fc*ε*RI complexes internalization and prevents all the hallmarks of MCs activation (red cross) such as calcium flux, degranulation, lipid mediators, and cytokine production.

This in vitro model of rapid MCs/IgE DS protocol has been reproduced in many studies and has consistently shown the inhibition of all of the MC activation hallmarks (Figure 3 and Figure 4) [45,53,54]. As shown in Figure 3 and Figure 4, compared to activated cells, desensitized cells had a diminished immediate release of β-hexosaminidase (Figure 3a), early and late TNF-α (Figure 3b), IL-6 production (Figure 3c), de novo synthesis of lipid mediators (Figure 3d), and calcium flux (Figure 3e) [53]. In vivo models of oral and intravenous rapid DS protocols were able to prevent passive systemic anaphylaxis (PSA). During DS with PSA mice models [45], there is an inhibition of body core temperature drop, release of serum mast cell protease-1 (mMCPT-1), and MC degranulation [45,54].

In addition, desensitized cells have shown impaired antigen/IgE/FcεRI complex internalization (Figure 4) [45,53]. This has been shown in different in vitro DS models, using different MCs (peritoneal), with different IgE antibodies and antigen doses, with the conclusion that complex internalization has a major role in DS [54].

Furthermore, challenging with the same antigen after being desensitized does not induce activation [53], however MCs can still be activated with different antigen stimulation, verifying the antigen specificity of DS (Figure 3e) [45,53]. This demonstrates that the mechanism of DS is an antigen-specific process and does not disable the IgE bounded FcεRI receptor for other antigens [45].

Furthermore, the treatment of desensitized cells with calcium ionophore A23187 or non-desenstizing antigen resulted in a high level of b-hexosaminidase release and calcium flux; indicating that desensitized cell mediators were not depleted and the non-IgE–mediated activation pathway was intact after DS [53]. In summary, the DS model is an antigen-specific desensitization and disables the specific response to one antigen but leaves the cell machinery unaffected, unlike non-specific DS. Even though the underlying mechanisms of DS are not fully understood, these in vitro and in vivo DS models can address hypotheses about its molecular pathway.

### 4.2. Proposed Mechanisms of Desensitization

#### 4.2.1. Ag/IgE-FcεRI Complex Mobility

Initial studies indicated that MC unresponsiveness after desensitization was due to internalization of FcεRI through progressive cross-linking at low antigen concentration, indicating that cell surface receptors were depleted before the next dose [62]. More recent studies have shown that antigen/IgE/FcεRI complexes’ internalization is impaired. Compared to activated cells where the majority are internalized, these complexes remain on the surface during DS (Figure 4) [45,53]. Also, the hyporesponsiveness after DS is not maintained because of the lack of free IgE bound receptor on the cell surface or the presence of an excess of soluble antigen since washed and re-sensitization cells remained desensitized. However, it is possible that the bound antigen is equilibrated in desensitized cells and the recovery of responsiveness is not due to the recycling of internalized IgE back to the cell surface [45].

In IgE mediated MC activation, the antigen valency and dose have been demonstrated to be key factors directly affecting FceRI behavior. Low doses of multivalent-antigen can create small clusters of Ag/IgE/FcεRI complexs which keep the receptors mobile on the cell surface. However, high doses or valency, result in larger cluster aggregates of receptors which induces their immobilization and subsequence internalization [63,64]. Whether the antigen/IgE/FcεRI complex mobility on the cell surface with a low dose of antigen could explain the impairment of receptor internalization during DS is unknown.

#### 4.2.2. ITAM/ITIM Counter Regulation

gp49B1 (LILRB4) receptors belong to the immunoglobulin (Ig) superfamily; which has two extracellular Ig-like domains and two ITIMs in its cytoplasmic domain. Colligation of gp49B1 (LILRB4) to crosslinked FcεRI will counter-regulate IgE-dependent MC activation in an ITIM dependent manner which has been evident in both in vitro and in vivo models. Mutation or deletion of gp49B1 (LILRB4) ITIM prevents the inhibition of MC activation. ITIM phosphorylation is required to recruit protein tyrosine phosphatases SH2-containing tyrosine phosphatase SHP 1, needed to dephosphorylate key signaling molecules, and consequently dampen the cellular response [65,66]. FcγRIIB, another inhibitory receptor that contains single ITIM in its cytoplasmic domain, recruits lipid phosphatase SH-2 containing inositol 5′ polyphosphatase (SHIP) which consequently decreases the levels of phosphatidylinositol 3,4,5-trisphosphate, a molecule generated by phosphatidylinositol 3-kinase upon cell activation [52,65]. It is likely that in the early steps of desensitization, the ITIM inhibitory pathway is dominant and during later steps overrides the activating signals. Nevertheless, further studies are needed to identify the ITIM inhibitory receptors and phosphatases regulating ITAM during DS.

Previous studies have shown that the inositol phosphatase SHIP can acts as a “gatekeeper” and negatively regulates MC degranulation for all antigen doses [63]. In addition, phospho-SHIP is rapidly recruited into the plasma membrane and colocalizes with FcεRI β receptors at both sub-optimal and supra-optimal doses which may explain the reduction of the degranulation response [63]. A time course study of phospho-SHIP colocalization with the FcεRI β receptor in RBL-2H3 cells has shown a peak at 2 min of FceRI crosslinking with a sub-optimal dose. In contrast, with optimal doses, phospho-SHIP colocalization was most notable at 5 min. SHIP phosphorylation is increased with non-optimal antigen dosing [63]. Therefore, multiple subthreshold doses of antigen during DS might result in the recruitment of SHIP which may play a critical role for tipping the balance between positive and negative signaling in the control of MC activation.

#### 4.2.3. Ca Channel Desensitization

Subthreshold antigen doses induce a small amount of intracellular calcium mobilization in MCs [45,53,54]. During DS, the sequential delivery of low antigen doses may create a continuous low level of intracellular calcium, which causes conformational changes of functional CRAC channels and other calcium related channels. These structural modifications in the receptors would block further calcium entry and signal transduction. However, challenging MCs after DS with a non-desensitizing antigen induces calcium flux (Figure 3e), proving that the DS is antigen-specific and the IgE mediated MC activation pathway is not disabled [53,54]. This fact could be explained by a membrane compartmentalization process which enables the exclusion of desensitized receptors exclusively [53].

#### 4.2.4. Actin Remodeling

Recent studies have shown the participation of actin cytoskeleton reorganization in calcium mobilization in many cells including MCs. Challenges with the same antigen after desensitization does not induce β-hexosaminidase release, calcium mobilization, or rearrangement of actin filaments [45,53]. The authors suggested that MC hyporesponsivness could be mediated by the highly stable remodeled actin cytoskeleton required in the compartmentalization of desensitized receptors, in addition to the surrounding signal transduction molecules. This aberrant remodeling of actin has been proposed as a negative regulator of calcium mobilization, preventing mediator release during DS [45].

## 5. Conclusions

**Figure 5:**
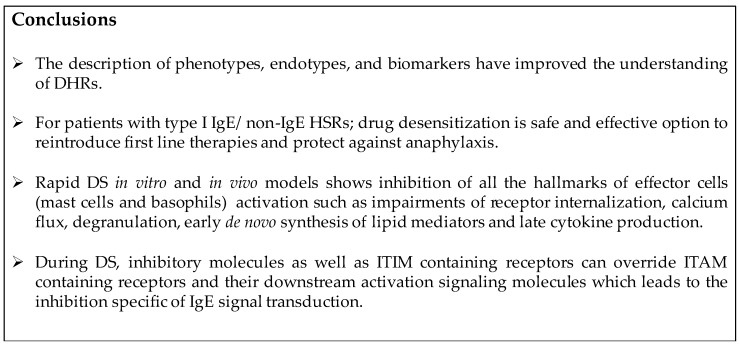
Conclusions.

The classical description of drug HSRs from Gell and Coombs is now complemented by the new understanding of phenotypes, endotypes, and corresponding biomarkers. This allows an expanded reaction classification, such as “cytokine storm-like reactions” to be recognized in HSRs to moAbs, oxaliplatin, and taxanes. New biomarkers from mast cells and other immune cell mediators (chymases, Carboxypeptidase A (CPA), Phospholipase A(PFA)) will be added in the future to allow for better categorization (Figure 5).

Desensitization is a revolutionary approach for the safe reintroduction of immunogenic drugs. Mast cells and basophils have long been known to be the cellular targets involved in desensitization; however the inhibitory mechanisms of desensitization are still being elucidated. DS takes advantage of inhibitory mechanisms which prevent activated mast cell signal transduction and pro-inflammatory mediator release.

Impairment of Ag/IgE/FεcRI complex internalization during desensitization can lead to these complexes remaining mobile on the cell surface. Low antigen doses administered incrementally along with actin remodeling could lead to an aggregation of these mobile complexes forming a secluded compartment of desensitized antigen specific receptors, Ca^2+^ channels, and signaling molecules. This compartment is specific to the antigen and excludes non-desensitized receptors (Figure 5).

Successful human DS protocols are based on in vitro IgE mast cell desensitization models and provide outstanding safety for all patients with severe allergic reactions in need of first line therapies. Future research will uncover the molecular pathway of in vitro desensitization which will permit more effective and safer human protocols.

## Figures and Tables

**Table:** (**a**)

Name of Medication: Penicillin			
Stock Solution	Voulum Per Stock Solution (mL)	Concentration (units/mL)	Total Dose Per Stock Solution (units)
1	30	1000	30,000
2	30	10,000	300,000
3	30	80,000	2,400,000
Target dose (units) = 1,296,700			

**Table:** 

Step	Stock Solution	Time (min)	Cumulative Time (min)	Voulum Given Per Step (mL)	Dose Given Per Step (units)	Cumulative Dose (units)	Fold Increase Per Step
1	1	15	15	0.1	100	100	0
2	1	15	30	0.2	200	300	2
3	1	15	45	0.4	400	700	2
4	1	15	60	0.8	800	1500	2
5	1	15	75	1.6	1600	3100	2
6	1	15	90	3.2	3200	6300	2
7	1	15	105	6.4	6400	12,700	2
8	2	15	120	1.2	12,000	24,700	1.875
9	2	15	135	2.4	24,000	48,700	2
10	2	15	150	4.8	48,000	96,700	2
11	3	15	165	1	80,000	176,700	1.67
12	3	15	180	2	160,000	336,700	2
13	3	15	195	4	320,000	656,700	2
14	3	15	210	8	640,000	1,296,700	2
Total time (h) = 3.5							

**Table:** (**b**)

Name of Medication: Obinutuzumab	
Target dose (mg)	750
Standard volume per bag (mL)	250
Final rate of infusion (mL/h)	80
Calculated target concentration (mg/mL)	3
Standard time of infusion (min)	187.5

**Table:** 

Bag	Volumen Per Bag (mL)	Concentration (mg/mL)	Total Dose Per Bag (mg)	Amount of Bag Infused (mL)
1	250	0.003	0.75	4.69
2	250	0.03	7.5	9.38
3	250	0.3	75	18.75
4	250	2.97632	744.08	250

**Table:** 

Step	Bag	Rate (mL/h)	Time (min)	Cumulative Time (min)	Volume Infused Per step (mL)	Dose Administrated with This Step (mg)	Cumulative Dose (mg)	Fold Increase Per Step
1	1	1.3	15	15	0.31	0.0009	0.0009	0
2	1	2.5	15	30	0.63	0.0019	0.0028	2
3	1	5	15	45	1.25	0.0038	0.0066	2
4	1	10	15	60	2.5	0.0075	0.0141	2
5	2	2.5	15	75	0.63	0.0188	0.0328	2.5
6	2	5	15	90	1.25	0.0375	0.0703	2
7	2	10	15	105	2.5	0.075	0.1453	2
8	2	20	15	120	5	0.15	0.2953	2
9	3	5	15	135	1.25	0.375	0.6703	2.5
10	3	10	15	150	2.5	0.75	1.4203	2
11	3	20	15	165	5	1.5	2.9203	2
12	3	40	15	180	10	3	5.9203	2
13	4	10	15	195	2.5	7.4408	13.3611	2.48
14	4	20	15	210	5	14.8816	28.2427	2
15	4	40	15	225	10	29.7632	58.0059	2
16	4	80	174.375	399.38	232.5	691.9941	750	2
Total time (h) = 6.66								

## Abbreviations

CPA	Carboxypeptidase A
DHR	Drug hypersensitivity reaction
DS	Desensitization
FcεRI	Fcε receptor I, high affinity Fc receptor for IgE
FcγRIIB	Fc receptors for IgG, type IIb
HSR	Hypersensitivity reaction
IgE	Immunoglobulin E
ITAM	Immune-receptor tyrosine-based activation motif
ITIM	Immunoreceptor tyrosine-based inhibitory motif
mBMMC	Mouse bone marrow-derived mast cell
MC	Mast cell
MrgprX2	Mas-related G-protein coupled receptor member X2
PFA	Phospholipase A
PSA	Passive systemic anaphylaxis
SHIP	SH-2 containing inositol 5′ polyphosphatase
SHP-1	SH-2 containing tyrosine phosphatase
